# Privies and Vaults

**Published:** 1875-04

**Authors:** 


Privies and Vaults.—It may very safely be concluded that where the population of any country town—depending upon wells for water—shows a considerable amount of diarrhœa, and also of typhoid fever, there is poison in the water-supply. It will be found that the contents of privies drain off into the wells, and contaminate the water used; for it is a fact, that the health of a population as regards diseases of the intestines, is very largely influenced by the freedom of the water from this most dangerous kind of contamination.

If any of our readers obtain any portion of their water from wells, let them see to it that no underground privy-vault exists within a distance of five rods, at least. If such a place is at hand, let it be filled up at once with clean fresh earth, and allow no more receptacles of the kind to be constructed. They are not only useless, but they are dangerous pests. They poison the air and the water, and induce a thousand diseases, and tens of thousands of deaths. Let the privy be situated on the top of the ground, with no excavation beneath it, and let sweet dry earth be sprinkled over the accumulations each day. A very little labor will secure entire purity in this regard. To dig a vault in these days of enlightenment is absolutely shameful. If anybody desires it, we will give precise directions for constructing an outhouse in a cheap, simple, and healthful manner, on the earth-closet plan,—the only sensible plan where no running water exists to carry off the excrement.

---

A sheep may get fat in a small meadow, and starve in a great desert.

